# Sensitivity Improvement via Differential Detection for Frequency-Locking Diamond Magnetometers

**DOI:** 10.3390/mi16101095

**Published:** 2025-09-26

**Authors:** Doudou Zheng, Jian Gao, Yang Li, Hui Wang, Yingjie Yang, Hao Guo, Huanfei Wen, Zhonghao Li, Jun Tang, Zongmin Ma, Jun Liu

**Affiliations:** 1 Department of Electronic Engineering, Taiyuan Institute of Technology, Taiyuan 030008, China; zhengddtit@163.com; 2 State Key Laboratory of Widegap Semiconductor Optoelectronic Materials and Technologies, North University of China, Taiyuan 030051, China; gaojian11_nuc@163.com (J.G.); liyang20160506@163.com (Y.L.); b20220621@st.nuc.edu.cn (H.W.); yangyingjie0926@163.com (Y.Y.); guohaonuc@163.com (H.G.); wenhuanfei@nuc.edu.cn (H.W.); lizh@nuc.edu.cn (Z.L.);; 3 State Key Laboratory of Extreme Environment Optoelectronic Dynamic Measurement Technology and Instrument, North University of China, Taiyuan 030051, China

**Keywords:** NV center, differential detection, frequency locking, sensitivity

## Abstract

The magnetic resonance frequency-locking technique is recognized as an effective approach for simultaneously improving the dynamic range, performance stability, and measurement precision of diamond nitrogen vacancy (NV)-center magnetometers. Nevertheless, insufficient research on sensitivity limits the overall performance of frequency-locking diamond magnetometers. In this paper, we propose a dual-magnetic-resonance-frequency-locking (MRFL) differential detection method. Theoretical and experimental results demonstrate that the scaling factor between the sensor output and the magnetic field is doubled compared with that under the single-MRFL method, and the proposed method also enables alternating current (AC) magnetic field detection. The proposed system exhibits a measurement range from −0.29 mT to 0.30 mT. Furthermore, a sensitivity of 0.56 nT/√Hz is achieved, representing a 58.2% improvement relative to that of the single-MRFL method. Our work provides a viable solution for accelerating the transition of frequency-locking diamond magnetometers from laboratory research to practical applications.

## 1. Introduction

In recent years, quantum magnetic sensors based on diamond nitrogen vacancy (NV) centers have made remarkable progress in the field of precision measurement, owing to their exceptional quantum-limited sensitivity [1,2], spatial resolution [3,4,5], and miniaturization potential [6,7,8]. However, for practical application requirements such as full magnetic navigation [9,10,11,12] and current detection [13,14,15,16], such sensors must simultaneously possess key performance indicators, including high precision [15,16], wide dynamic range [17,18,19], high performance stability [16] and high sensitivity. Achieving these indicators is of great significance for their reliable operation in dynamic and complex environments.

Magnetic resonance frequency-locking (MRFL) technique, which has emerged as a pivotal solution for achieving the aforementioned comprehensive performance enhancements [15,16,17,18,19,20,21], enables exceptional measurement precision of 0.061% [15], a dynamic range exceeding 11 mT [17], and robust temperature stability with a variation of ±0.3% over the temperature range from −40 °C to 85 °C [16]. This approach establishes a closed-loop system through lock-in signal processing via a proportional–integral–derivative (PID) controller. When external magnetic field variations induce electron spin resonance detuning, the system generates a PID output signal proportional to the frequency shift. The PID output signal dynamically adjusts the microwave excitation frequency through real-time feedback, maintaining continuous magnetic resonance frequency locking. Consequently, MRFL enables both real-time magnetic field tracking and significant expansion of sensor dynamic range and measurement span. Furthermore, multi-resonance frequency locking schemes enable simultaneous monitoring of both magnetic field and temperature responses, effectively suppressing thermal drift while enhancing immunity to interference [22]. However, for high-performance diamond magnetometers to be practically applied, overcoming sensitivity limitations in multi-MRFL systems is essential.

Here, we propose a dual-resonance-frequency-locking differential detection method to achieve high-sensitivity magnetic field detection. By simultaneously acquiring dual-channel PID outputs in real-time and applying differential processing, we obtain the magnetic field-to-voltage transfer coefficient. The results show that, compared with the traditional single-MRFL method, the dual-MRFL scheme achieves a two-fold enhancement in the scaling factor between the sensor output and the magnetic field for frequency-locked magnetometers, while improving the magnetometer’s sensitivity to 0.56 nT/√Hz. Notably, this method exhibits excellent scalability, by which the sensitivity of vector magnetic field measurement can be improved through the simultaneous locking of four pairs of resonance signals. This study provides a new technical solution for the engineering applications of diamond magnetometers in fields such as precision navigation and high-current detection.

## 2. Principle

**Figure 1:**
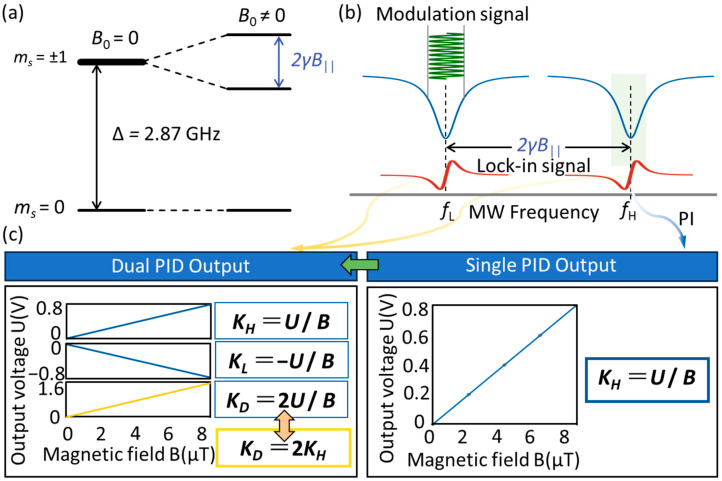
(**a**) Ground-state triplet energy levels of the NV center in diamond. (**b**) ODMR spectrum and lock-in signal under an external magnetic field. (**c**) Comparison of magnetic scaling factors for single-MRFL versus dual-MRFL differential detection methods.

The magnetic field detection capability of NV centers stems from their triplet ground state, as depicted in Figure 1a. At zero external magnetic field, the *m_s_* = +1 and *m_s_* = −1 states are degenerate, and the zero-field splitting between the *m_s_* = 0 state and *m_s_* = ±1 states is 2.87 GHz. Under an external magnetic field, the *m_s_* = ±1 states undergo Zeeman splitting [23]. The resulting energy splitting *f* between the *m_s_* = +1 and *m_s_* = −1 states scales linearly with the magnetic field projection along the NV symmetry axis *B*, following the Larmor precession formula *f* = 2γ*B*. Here, γ ≈ 28 MHz/mT represents the electronic gyromagnetic ratio of the NV center.

As a classical method for magnetic field detection, optically detected magnetic resonance (ODMR) relies on a core principle that involves exciting the Zeeman energy level resonant transitions of NV centers from the *m_s_* = 0 state to the *m_s_* = ±1 states via microwave frequency sweeping. By utilizing the photoluminescent properties of NV centers to detect transition signals, ODMR spectra are obtained. The magnitude of the magnetic field to be measured can be derived by extracting the frequency difference between the two resonance peaks in the spectrum, and performing inversion calculations in conjunction with the Larmor precession formula *f* = 2γ*B*. To achieve real-time dynamic detection of magnetic fields and suppress low-frequency 1/*f* noise interference, the frequency modulation method is developed based on traditional ODMR technology, as shown in Figure 1b. Specifically, after the microwave carrier is modulated by a reference signal and radiated to the NV centers, a lock-in amplifier coherently demodulates the ODMR signal, yielding the lock-in signal. The point of maximum slope of the lock-in signal corresponds to the resonance frequency, and its zero-point voltage offset exhibits a linear relationship with magnetic field variations. By utilizing the pre-calibrated voltage-frequency scaling factor, real-time magnetic field measurement can be realized. However, this scaling factor exhibits temporal drift, which in turn requires regular calibration to maintain the accuracy of the detection.

The lock-in signal is fed into a PID closed-loop feedback system, which dynamically tunes the microwave frequency to lock the lock-in signal to its zero point, as shown in the right panel of Figure 1c. Then, the variation in the microwave source’s output frequency is directly reflected in real time via the PID output signal *U*. By combining the Larmor precession relation with the frequency-to-voltage conversion, we derive the scaling factor *K*_H_ = *U*/*B* that converts the PID output signal into magnetic field strength, enabling real-time magnetic field measurement. In this paper, we propose a dual-MRFL differential detection method to improve the sensitivity of magnetic field detection, where the lower and upper resonance frequencies are denoted as *R*_L_ and *R*_H_, respectively. When the magnetic field changes, the PID output signals corresponding to the two resonance frequencies exhibit an opposite trend, enabling the two microwave generators to accurately track *R*_L_ and *R*_H_, whose corresponding scaling factors for the two resonance frequencies are, respectively, denoted as *K*_H_ = *U*/*B* and *K*_L_ = −*U*/*B*. By performing a differential operation on the dual-PID output signals, we obtain the differential scaling factor *K*_D_ = 2*U*/*B*. Compared with that of the single-MRFL scheme, the scaling factor between the sensor output and magnetic field for the PID feedback differential detection is doubled, as shown in the left panel of Figure 1c.

The carrier signal generated by the microwave source has a frequency of *f_c_*. After applying the modulation signal *m*(*t*), the instantaneous output frequency *f_i_*(*t*) of the microwave source is given by Equation (1).

(1) $$f_{i}\left(t\right)=f_{c}+K_{VCO}m\left(t\right)$$

Here, *K*_VCO_ denotes the voltage-to-frequency conversion gain (termed modulation sensitivity) of the voltage-controlled oscillator (VCO), with units of Hz/V, characterizing the frequency deviation per unit modulation voltage. The term $K_{VCO}m\left(t\right)$ denotes the instantaneous frequency deviation.

The corresponding instantaneous phase $\theta_{i}\left(t\right)$ is expressed as:

(2) $$\theta_{i}\left(t\right)=2\pi f_{c}t+2\pi K_{VCO}\int_{0}^{t}m\left(t\right)dt$$

The resulting frequency-modulated (FM) signal is therefore given by:

(3) $$x_{FM}\left(t\right)=A_{c}cos\left[2\pi f_{c}t+2\pi K_{VCO}\int_{0}^{t}m\left(t\right)dt\right]$$

here, *A_C_* denotes the carrier amplitude.

When the modulation signal is a DC signal, i.e., *m*(*t*) = *A_m_*, the instantaneous microwave output signal is given by:

(4) $$\begin{array}{cc} x_{FM}\left(t\right) & =A_{c}cos\left[2\pi f_{c}t+2\pi K_{VCO}A_{m}t\right]\\ & =A_{c}cos\left[2\pi \left(f_{c}+K_{VCO}A_{m}\right)t\right] \end{array}$$

The system employs a PID controller to achieve precise microwave frequency locking onto the target resonance peak. The controller generates a control signal *u*(*t*) based on the error signal *e*(*t*), which is the deviation between the zero point of the lock-in signal and its actual amplitude. The control signal *u*(*t*) is composed of a linear combination of the proportional (*P*) term, integral (*I*) term, and derivative (*D*) term of the error:

(5) $$u\left(t\right)=K_{P}e\left(t\right)+K_{I}\int e\left(t\right)dt+K_{D}\frac{de\left(t\right)}{dt}$$

**Figure 2:**
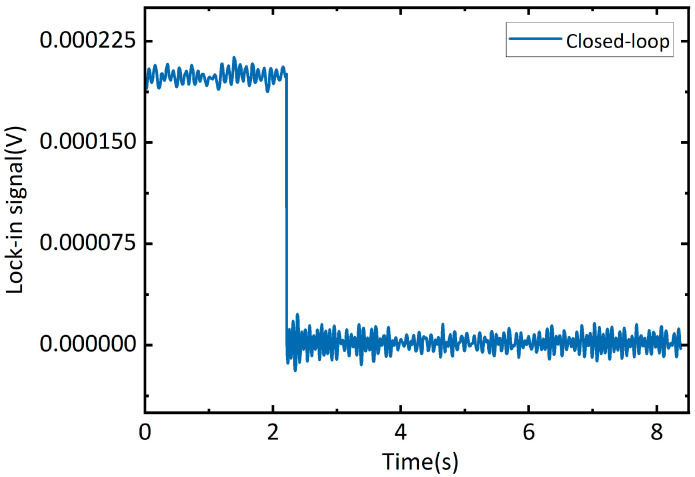
Step response of the zero-crossing output for the lock-in signal.

This control signal *u*(*t*) drives the microwave source, stabilizing its output near the resonance peak through frequency adjustment. Following experimental parameter tuning, the PID gains are set as follows: proportional gain *K_P_* = 0.4 and integral gain *K_I_* = 0.6 that primarily governs the locking rate. Experimental observations demonstrate that introducing the derivative gain *K_D_* causes rapid loss of lock. Consequently, the derivative term is disabled in the final configuration (*K_D_* = 0). The output of the PID controller serves as the DC modulation signal *m*(*t*) input to the microwave source, whose frequency is determined by Equation (4). With the above parameter configuration of *K_P_* = 0.4, *K_I_* = 0.6, *K_D_* = 0, the system successfully locks the lock-in signal from an initial deviation of 0.00018 V to the target of 0 V, as shown in Figure 2.

## 3. Experimental Setup

**Figure 3:**
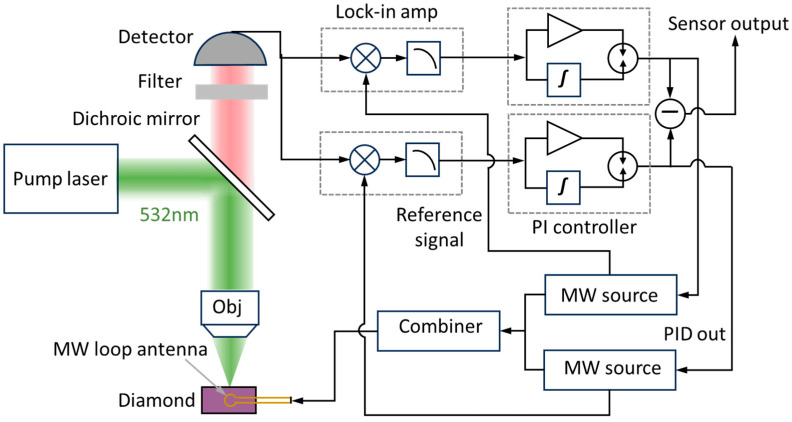
The schematic diagram of the dual-MRFL differential detection system.

The schematic diagram of the dual-MRFL differential detection system is shown in Figure 3. The optical path employs a confocal system. Under the combined excitation of a microwave field, a magnetic field, and a 532 nm laser, the NV centers in diamond generate ODMR fluorescence signals. Notably, the experiment employed a single-crystal CVD diamond sample with dimensions of 3.5 mm × 3.5 mm × 0.2 mm and an initial nitrogen concentration of approximately 100 ppm. The sample was first irradiated with 10 MeV electrons for approximately 4 h. For subsequent processing, the sample was annealed at 600 °C for 1 h, followed by a 30 min hold, and finally heated to 800 °C for a 4 h vacuum anneal to form NV centers. After passing through a 600–800 nm filter that removes 532 nm excitation light and background stray light, the fluorescence signals are collected and photoelectrically converted by a photodetector.

Two N5181B signal generators (Keysight Technologies, Santa Rosa, CA, USA) output microwave signals corresponding to their respective target ODMR peaks, each with an output power of 20 dBm. These signals are combined through a microwave combiner for multi-frequency synthesis and fed into a loop antenna surrounding the diamond. Simultaneously, reference signals are generated by these microwave sources. One is used to modulate the carrier frequency, while the other is fed to a lock-in amplifier for demodulation. Modulation parameters are configured to a modulation frequency of 500 Hz with a 3 MHz deviation.

The HF2LI lock-in amplifier (Zurich Instruments, Zurich, Switzerland) performs dual-core functions. It synchronously acquires fluorescence signals detected by the photodetector, utilizes its built-in multiplier to mix modulated fluorescence signals with a reference signal, and extracts lock-in signals via a low-pass filter module. Subsequently, its integrated PID controller locks the lock-in signal at the zero point by calculating the PID output from the error signal, shift and scale. The error signal quantifies the deviation between the microwave frequency and the ODMR frequency, specifically zero at resonance, positive during high-frequency detuning, and negative during low-frequency detuning. Following PI compensation, the resultant PID output is fed back to the corresponding microwave source’s external modulation port, establishing a closed-loop frequency tracking system, while the sensor’s final output is defined as the difference between both independent PID output signals.

## 4. Results and Discussion

### 4.1. Scaling Factor

**Figure 4:**
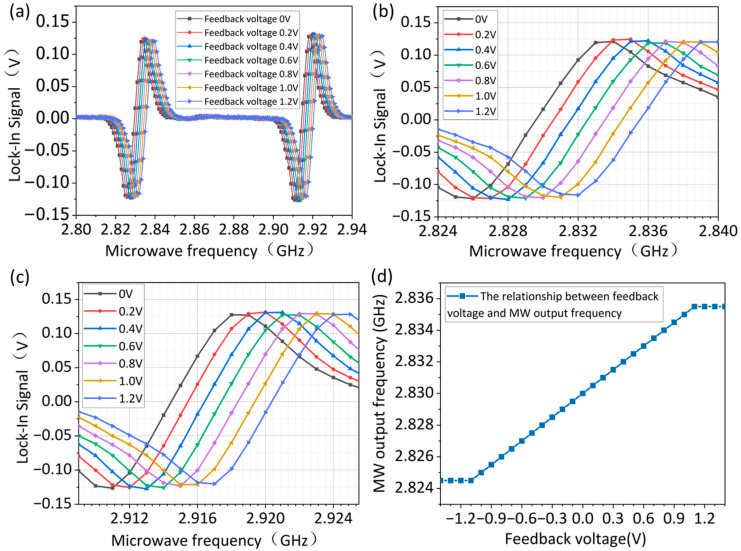
(**a**) Lock-in signals under different feedback voltages, (**b**) corresponds to the left resonance peak, while (**c**) corresponds to the right resonance peak. (**d**) The scaling factor between the microwave output frequency and the feedback voltage.

Figure 4 illustrates the correspondence between the PID output (i.e., feedback voltage) and the microwave frequency output. In the experiment, a bias magnetic field of 2.6 mT was applied perpendicular to the (100) crystal face of the diamond. The first frequency modulation (FM) channel was configured with a modulation frequency of 500 Hz and a modulation deviation of 3 MHz to acquire the lock-in signal corresponding to a feedback voltage of 0 V. After adjusting the external modulation deviation of the second FM channel to 5 MHz, Figure 4a shows the lock-in signals over the 2.80–2.94 GHz range under DC modulation voltages of 0.2 V, 0.4 V, 0.6 V, 0.8 V, 1.0 V, and 1.2 V. The evolution of the left resonance peak is shown in Figure 4b, while the characteristics of the right resonance peak are presented in Figure 4c.

As can be seen from the experimental results, with the increase in the feedback voltage, the output frequency of the microwave source changes linearly. When the feedback voltage is 1 V, the change in the microwave output frequency is exactly equal to the second channel’s modulation deviation of 5 MHz, which is consistent with the theoretical result of Equation (4). Figure 4d further quantifies this linear relationship, establishing the scaling factor between the PID output and the microwave frequency as 5 MHz/V. It should be noted that the splitting of the demodulated signal in Figure 4a is not caused by changes in the magnetic field, but stems from the fact that the abscissa represents the microwave carrier frequency, and the observed splitting originates from the actual change in the output frequency of the microwave source.

**Figure 5:**
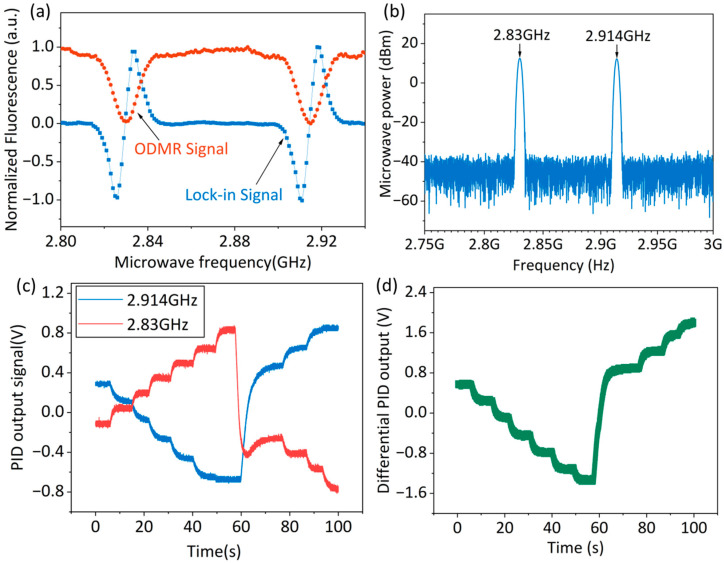
(**a**) The ODMR and lock-in signals under a magnetic field applied perpendicular to the (100) crystal face of the diamond. (**b**) The 2.83 GHz and 2.914 GHz outputs on the spectrum analyzer after passing through the mixer. (**c**) The step responses of the demodulation signals at 2.914 GHz and 2.83 GHz under different magnetic field changes. (**d**) Differential output results.

Figure 5a shows the normalized fluorescence intensity as a function of microwave frequency. The red curve represents the ODMR signal, exhibiting two distinct resonance peaks at 2.83 GHz and 2.914 GHz, respectively. The solid blue curve denotes the lock-in signal, whose waveform approximates the reciprocal of the ODMR signal [24]. Figure 5b presents the verification results of the combined microwave signal obtained using a spectrum analyzer, revealing peak signals at 2.83 GHz and 2.914 GHz. After attenuation by the combiner, the peak power of the signal fed to the antenna is approximately 17 dBm.

Figure 5c illustrates the time-dependent variations in the two PID output signals over the range of 0–100 s during manual magnetic field adjustment. The solid blue and red curves represent the lock-in signals at 2.914 GHz and 2.830 GHz, respectively. Before 60 s, the continuously increasing magnetic field caused the 2.914 GHz resonance peak to shift rightward, resulting in a gradual rise in its PID output with increasing field strength. Conversely, the 2.830 GHz resonance peak shifted leftward, causing its PID output to decrease gradually with increasing magnetic field. At 60 s, upon directional adjustment of the magnetic field, both PID outputs showed abrupt transients. Subsequently, as the magnetic field increased again, the PID outputs for the 2.914 GHz and 2.830 GHz channels, respectively, rose and fell. Figure 5d displays the differential output of the two PID signals, whose amplitude doubles the single-channel response for identical magnetic field changes. It should be emphasized that the long response time of the PID output signal observed in Figure 5c,d is primarily due to the manual current input for adjusting the Helmholtz coil’s magnetic field and the use of an oscilloscope to display the output. To address this issue, the real-time PID output display module may be integrated into the lock-in amplifier system in subsequent work.

**Figure 6:**
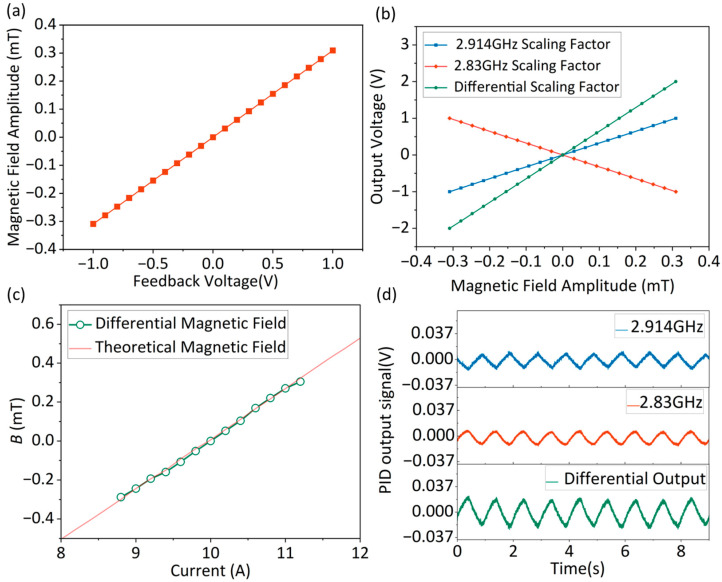
(**a**) Relationship between magnetic field amplitude and feedback voltage. (**b**) Scaling factor between sensor output and magnetic field amplitude under single-MRFL and dual-MRFL differential detection methods. (**c**) Experimental and theoretical magnetic field variation output during Helmholtz coil current variation. (**d**) Sensor output results under an AC magnetic field with a frequency of 1 Hz.

Using a single resonance peak’s feedback voltage for analysis, Figure 4d demonstrates a 5 MHz/V scaling factor between the feedback voltage and microwave output frequency. Combining this with the Larmor precession formula, we derive the fundamental feedback voltage-to-magnetic field ratio as 5/28 mT/V. Considering the magnetic field perpendicular to the (100) crystal face of the diamond, a √3-fold correction factor must be introduced into the actual corresponding relationship between the external magnetic field and the voltage, resulting in a final value of 0.309 mT/V, as shown in Figure 6a. Figure 6b presents the magnetic field scaling factors for the PID output voltage at the 2.830 GHz and 2.914 GHz resonance peaks. The red and blue curves correspond to scaling factors of 3.24 V/mT and −3.24 V/mT, respectively, while the green curve shows a 6.48 V/mT scaling factor from the differential processing of the dual-channel signals. Figure 6c is used to verify the accuracy of the differential experimental results. A 2.6 mT bias magnetic field corresponding to a coil current of 10 A was applied to the NV centers, to achieve the splitting of the 2.83 GHz and 2.914 GHz peaks in the ODMR spectrum. The green curve shows the magnetic field–current relationship measured via the dual-MRFL differential detection method during coil current variations from 8.8 A to 11.2 A, while the red curve represents the calibration result of the coil magnetic field versus applied current. It can be seen that the experimental results match the theoretical values, with an output range of approximately −0.29 to 0.30 mT. Figure 6d displays the PID output for a 2.9 μT and 1 Hz sinusoidal magnetic field, confirming the differential detection method’s applicability to AC signal measurements.

### 4.2. Sensitivity

**Figure 7:**
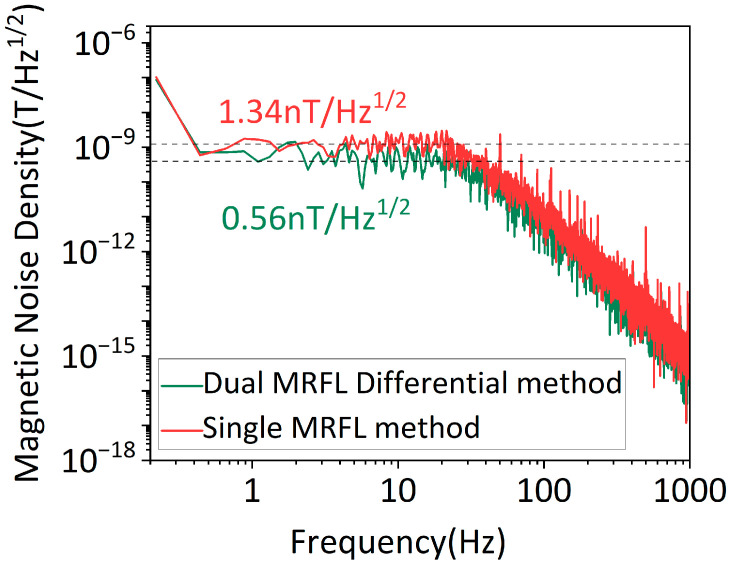
Noise spectral densities for single-MRFL and dual-MRFL differential detection methods.

Under zero-external magnetic field conditions, amplitude spectral density (ASD) measurements of the sensor output signal were performed and converted to magnetic field detection sensitivity using Equation (6) [25].

(6) $$\eta =\frac{\mathrm{ASD}}{k}$$

where *k* represents the scaling factor between the sensor output voltage and the magnetic field. Based on the results from Figure 6b, the scaling factors for single-MRFL and dual-MRFL differential detection methods are 3.24 V/mT and 6.48 V/mT, respectively. Therefore, the corresponding magnetic field detection sensitivities calculated are 1.34 nT/√Hz and 0.56 nT/√Hz, respectively, as shown in Figure 7. These sensitivity values are average values within the frequency range of 0.5 Hz to 10 Hz, which indicates that the sensitivity under the dual-MRFL differential detection method is reduced to 0.42 times that of the single-MRFL method, representing an improvement of 58.2%.

As summarized in Table 1, recent years have witnessed considerable efforts in developing frequency-locking diamond magnetometers. However, most existing studies have primarily focused on improving dynamic range and detection precision, with their sensitivity limited to the nT level. In contrast, the dual-MRFL differential detection method proposed in this paper achieves a sensitivity of 0.56 nT/√Hz, offering valuable reference for sensitivity enhancement of frequency-locking diamond magnetometers.

## 5. Conclusions

In summary, we proposed a dual-MRFL differential detection method to enhance the sensitivity of frequency-locking diamond magnetometers. By establishing a dual-channel PID feedback model and combining it with experimental verification, we demonstrated that this method effectively increases the scaling factor between the sensor output and the magnetic field, thereby improving the sensitivity to 0.56 nT/√Hz, which represents a 58.2% improvement compared with that of the single-MRFL method. The technique will provide critical support for improving the overall performance of frequency-locking diamond magnetometers, thereby accelerating their transition from laboratory research to practical applications.

## Figures and Tables

**Table 1:** Performance comparison of frequency-locking diamond magnetometers.

Groups	Years	Dual PID	Sensitivity
H. Clevenson et al. [17]	2018	No	1 nT/√Hz
Y. Hatano et al. [16]	2022	Yes	4.7 nT/√Hz
H. Kumar et al. [18]	2024	No	10 nT/√Hz
Q. Liu et al. [15]	2024	Yes	3.7 nT/√Hz
Our work	2025	Yes	0.56 nT/√Hz

## Data Availability

Data is contained within the article.
